# Everolimus-induced human keratinocytes toxicity is mediated by STAT3 inhibition

**DOI:** 10.1186/1756-9966-32-83

**Published:** 2013-10-25

**Authors:** Kazuhiro Yamamoto, Atsushi Uda, Akira Mukai, Kazuhiko Yamashita, Manabu Kume, Hiroo Makimoto, Toshinori Bito, Chikako Nishigori, Takeshi Hirano, Midori Hirai

**Affiliations:** 1 Department of Pharmacy, Kobe University Hospital, 7-5-2 Kusunoki-cho, Chuo-ku, Kobe 650-0017, Japan; 2 Division of Dermatology, Department of Internal Related, Kobe University Graduate School of Medicine, 7-5-2 Kusunoki-cho, Chuo-ku, Kobe 650-0017, Japan; 3 Division of Pharmacokinetics, Department of Biochemistry and Molecular Biology, Kobe University Graduate School of Medicine, 7-5-2 Kusunoki-cho, Chuo-ku, Kobe 650-0017, Japan

**Keywords:** Everolimus, Skin toxicity, Skin rush, Hand–foot skin reaction, Keratinocyte, STAT3

## Abstract

**Background:**

Mammalian target of rapamycin (mTOR) inhibitors are associated with dermatological adverse events. The chief aim of this study was to examine the relation between the signal transducer and activator of transcription 3 (STAT3) protein and the dermatological adverse events associated with the mTOR inhibitor everolimus.

**Methods:**

We evaluated the effects of STAT3 activity and related signal transduction activities on everolimus-induced cell growth inhibition in the human keratinocyte HaCaT cell line via a WST-8 assay, and on signal transduction mechanisms involved in everolimus treatments via a western blot analysis. Apoptosis was evaluated using an imaging cytometric assay.

**Results:**

The cell growth inhibitory effects of everolimus were enhanced by stattic or STA-21, which are selective inhibitors of STAT3, treatment in HaCaT cells, although such effects were not observed in Caki-1 and HepG2 cells. Phosphorylation at tyrosine 705 of STAT3 was decreased by treatment with everolimus in a dose-dependent manner in HaCaT cells; in contrast, phosphorylation at serine 727 was not decreased by everolimus, but slightly increased. Furthermore, we found that pretreatment of p38 MAPK inhibitor and transfection with constitutively active form of STAT3 in HaCaT cells resisted the cytostatic activity of everolimus.

**Conclusions:**

These findings suggest that STAT3 activity may be a biomarker of everolimus-induced dermatological toxicity.

## Background

Cancer chemotherapy made dramatic progress with the advent of molecular target drugs. Development of these molecules for the treatment of various types of cancer is expected in the future. However, serious adverse events were observed with continuous treatment of cancer by molecular target drugs that are considered as more safe therapeutic options. In particular, dermatological adverse events, sometimes termed as “hand–foot skin reaction”, occur at an exceptionally high frequency during the use of specific drugs thus leading to interruption of therapy or depression in quality of life [1-4]. These dermatological side effects are differentiated from dermatitis resulting from cytotoxic anticancer agents, e.g., 5-fluorouracil and drugs in the taxane group, and they exhibit a characteristic pathological model [3]. Furthermore, clinicopathological findings have shown that these dermatological side effects are due to deficiency in epidermal cell growth [5]. In addition, these effects are present in a localized area of the body [5]. Moreover, these side effects are correlated with therapeutic effects [3-5]. Although they pose a critical issue for patients receiving targeted molecular therapy, the pathogenic mechanisms underlying these side effects remain unclear.

Mammalian target of rapamycin (mTOR) inhibitors (rapamycin, everolimus, and temsirolimus) are a new class of anticancer drugs with a novel mechanism of action. These compounds inhibit the proliferation and growth of a wide spectrum of tumor cell lines by inhibiting signal transduction from the phosphatidylinositol 3-kinase (PI3K)/protein kinase B (Akt)/mTOR pathway [6]. The potential benefits of mTOR inhibitors have not been fully realized because of the various side effects of these drugs. The incidence of dermatitis in sirolimus-treated patients is in the range of 13–46% in different studies [7-9]. An effective breakthrough regarding the cutaneous side effects of treatment with mTOR inhibitors remains crucial.

The signal transducer and activator of transcription (STAT) signaling pathways are activated in response to cytokines and growth factors [e.g., epidermal growth factor (EGF) and vascular endothelial growth factor (VEGF)] [10,11]. STAT3 exerts widespread effects via the transcriptional upregulation of genes encoding proteins involved in cell survival, cell–cycle progression, and homeostasis [12,13]. Moreover, transcription mediated by phosphorylated STAT3 (pSTAT3) controls several genes of the apoptotic pathway, including the bcl family and inhibitors of apoptosis family of genes [14]. A recent study reported that STAT3 is the main factor in the molecular control of cutaneous homeostasis [15]. Inhibition of STAT3 (as an important factor in the formation of skin lesions) has the potential to be one of the pathogenic mechanisms underlying the dermatological side effects induced by treatment with molecular target drugs.

In the present study, we investigated the effects of STAT3 and related mechanisms on everolimus-mediated cell growth inhibition in human epidermal keratinocyte cell lines. Our findings suggest that STAT3 activity in keratinocytes may be a biomarker of everolimus-induced dermatological events.

## Materials and methods

### Chemicals

**Figure 1:**
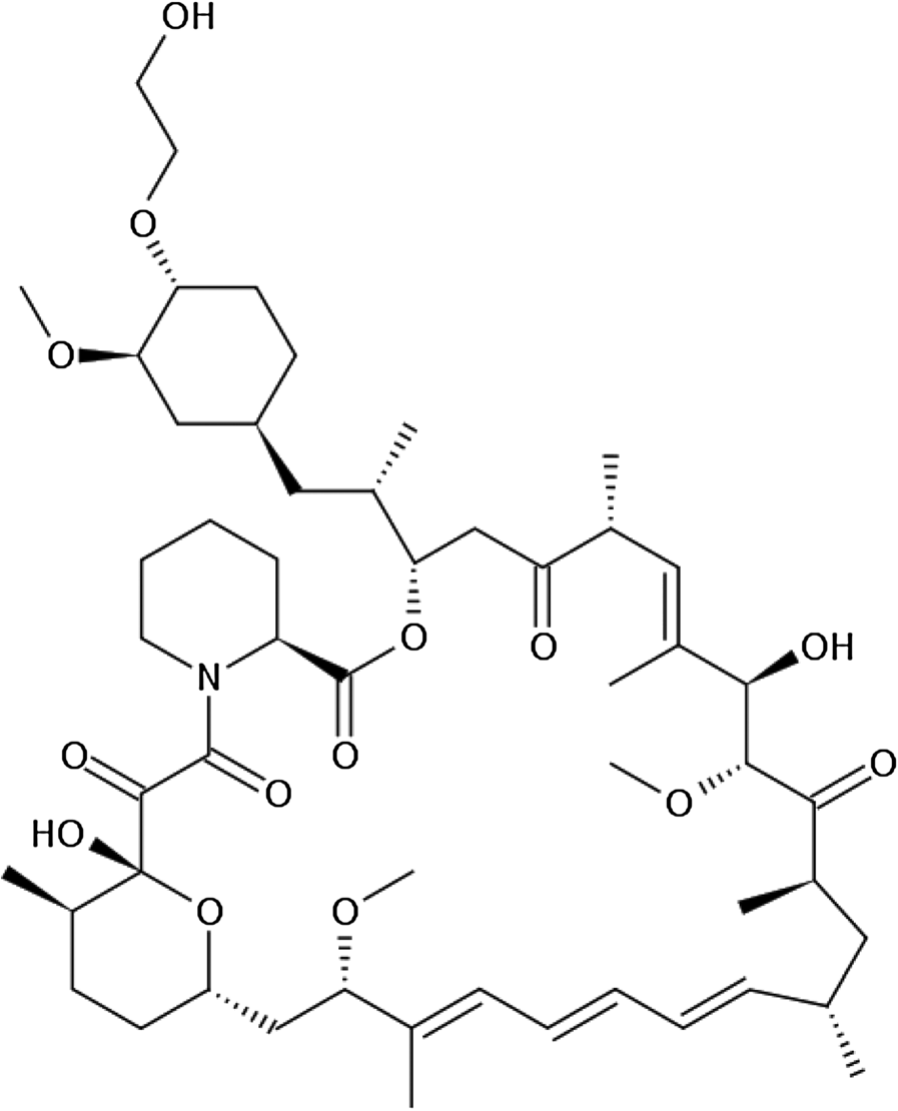
Chemical structure of everolimus.

Everolimus (Figure 1), a derivative of sirolimus and an mTOR inhibitor, was purchased from Sigma-Aldrich Chemical, Co. (St Louis, MO, USA). Stattic, a small-molecule inhibitor of STAT3 activation [16], was purchased from Enzo Life Sciences, Inc. (Farmingdale, NY, USA). STA-21, a STAT3 inhibitor [17], was purchased from Santa Cruz Biotechnology (Santa Cruz, CA, USA). Z3, an inhibitor of the autophosphorylation of Janus kinase 2 (JAK2) [18], was obtained from Calbiochem (Darmstadt, Germany). SB203580, a specific blocker of p38 mitogen-activated protein kinase (MAPK) activity, and SP600125, a selective and reversible inhibitor of the c-Jun N-terminal kinase 1 (JNK1), JNK2, and JNK3, were obtained from Cayman Chemical Company (Ann Arbor, MI, USA). U0126, a selective inhibitor of mitogen-induced extracellular kinase 1 (MEK1) and MEK2, was purchase from Cell Signaling Technology, Inc. (Boston, MA, USA).

### Antibodies

Rabbit anti-phosphorylated (anti-phospho)-STAT3 at tyrosine 705 (Tyr705) and serine 727 (Ser727), mouse anti-STAT3 antibodies, rabbit anti-phospho-extracellular signal-regulated kinase (Erk) 1/2, rabbit anti-Erk 1/2 antibodies, rabbit anti-phospho-p38 MAPK, rabbit anti-p38 antibodies, anti-phospho-S6 kinase (Thr389) and anti-p70 S6 kinase antibodies were purchased from Cell Signaling Technology. Mouse anti-phospho-JNK and rabbit anti-JNK antibodies, as well as anti-mouse HRP-conjugated IgG, anti-rabbit HRP-conjugated IgG, and anti-rabbit FITC-conjugate IgG, were purchased from Santa Cruz Biotechnology. A rabbit anti-β-actin antibody was obtained from Sigma-Aldrich.

### Cells and cell culture

HaCaT cells, the human immortalized keratinocyte cell lines, were kindly provided by Professor Norbert Fusenig (German Cancer Research Centre, Heidelberg, Germany) [19]. HepG2 cells, the human hepatocarcinoma cell lines, were purchased from JCRB (Osaka, Japan). HaCaT and HepG2 cells were maintained in Dulbecco’s Modified Eagle’s Medium (DMEM; Sigma-Aldrich) supplemented with 10% heat-inactivated fetal bovine serum (lot. No. 9866 J; MP Biomedicals, Solon, OH, USA), 100 units/mL of penicillin, and 100 μg/mL streptomycin (Life Technologies, Carlsbad, CA, USA). Caki-1 cells, the human renal cell carcinoma cell lines, were purchased from JCRB. Caki-1 cells were maintained in Eagle’s Minimum Essential Medium (EMEM; Sigma-Aldrich) supplemented with 10% heat-inactivated fetal bovine serum, 100 units/mL of penicillin, and 100 μg/mL streptomycin, similar to the HaCaT culture medium. Each cell line was seeded into culture flasks, grown in a humidified atmosphere of 5% CO_2_ and 95% air at 37°C, and subcultured with 0.05% trypsin/0.02% EDTA (Life Technologies).

### WST-8 colorimetric assay

The effects of various signal transduction inhibitors and transfection with expression plasmids on the everolimus-mediated cell growth inhibition in HaCaT cells were evaluated via the WST-8 assay using the Cell Counting Kit-8 (Dojindo Laboratories, Kumamoto, Japan) as described previously [20-22]. Cells (2 × 10^3^/well) were seeded onto 96-well plates and precultured for 24 h. The medium was exchanged for medium containing everolimus at various concentrations after pretreatment with signal transduction inhibitors at several concentrations, for appropriate term, followed by incubation for 48 h at 37°C. The culture medium was replaced with a medium containing a WST-8 reagent for 3 h and the absorbance in the well was determined at 450 nm with a reference wavelength of 630 nm using a microplate reader (FLUOstar OPTIMA, BMG LABTECH, Ltd., Germany).

### Apoptosis assay

Apoptosis-mediated cell death was examined in HaCaT cells by a double-staining method using a FITC-labeled Annexin V/propidium iodide (PI) apoptosis detection kit (BD Biosciences, San Jose, CA, USA) according to the manufacturer’s instructions. In brief, control, everolimus-treated, and stattic-treated cells were washed in phosphate-buffered saline (PBS) twice and incubated with PBS containing FITC-conjugated Annexin V and PI dyes for 30 min at 37°C. After cells were washed in PBS twice, they were incubated with PBS containing 10 μM Hoechst 33258 and 4% paraformaldehyde for 30 min at 37°C. The externalization of phosphatidylserine and the permeability to PI were evaluated using an IN Cell Analyzer 2000 (GE Healthcare UK Ltd, Buckinghamshire, UK). Cells in early stages of apoptosis were positively stained with Annexin V, whereas cells in late apoptosis were positively stained with both Annexin V and PI.

### Western blotting

Western blotting was performed as described previously [6]. Proteins in the total cell lysate were extracted from cells treating to each buffer with Cell Lysis Buffer (Cell Signaling Technology) in addition to 1 mM dithiothreitol, 1 mM phenylmethylsulfonyl fluoride, and 5 μg/mL leupeptin. Proteins were separated using 7.5 or 12% sodium dodecyl sulfate-polyacrylamide gel (SDS-PAGE) electrophoresis and electrotransferred to a polyvinylidene difluoride membrane (Hybond-P membrane; GE Healthcare). Subsequently, the blot was blocked in a solution of wash buffer (10 mM Tris, pH 7.5, 150 mM NaCl, and 0.05% Tween-20) containing 5% skim milk. The membrane was soused in wash buffer containing specific primary antibodies overnight, followed by incubation with horseradish peroxidase-conjugated secondary antibodies for 1 h. Antibody-bound proteins were visualized by treating the membrane with the enhanced ECLTM Prime Western Blotting Detection Reagent (GE Healthcare) prepared immediately before detection. Finally, blot images were acquired using ChemiStage 16-CC (KURABO Industries Ltd., Osaka, Japan). Wherever indicated, the membranes were stripped and reprobed with another antibody.

### Plasmid construction

Constitutively active STAT3 (STAT3C) mammalian expression plasmids were kindly provided by Professor Miyajima (University of Tokyo, Tokyo, Japan) [23]. Tyrosine 705 deficient STAT3 (STAT3-Y705F) mammalian expression plasmids were kindly provided by Darnell (Addgene plasmid #8709) [24]. STAT3C and STAT3-Y705F constructs were transformed into DH-5α competent cells and plasmid DNA was extracted using the QIAGEN® Plasmid Midi Kit (QIAGEN K.K, Tokyo, Japan). Extracted plasmids were purified to a grade appropriate for cell culture using phenol and chloroform and stocked at 1 μg/μL in a freezer until experimental use.

### Transient transfection

Transient transfection of cell lines with expression vectors was performed using the Lipofectamine LTX transfection reagent (Life Technologies) according to the manufacturer’s protocol. In brief, cells were grown in 96-well culture plates until they reached ~90% confluence. The culture medium was replaced with serum-free Opti-MEM (Life Technologies) and cells were transfected with the DNA–lipofectamine complex. HaCaT cells were transiently transfected with 0.1 μg/well of plasmid in 96-well plates.

### Immunofluorescence imaging and cytometric analysis

Transfected HaCaT cells were fixed with 4% paraformaldehyde for 15 min at room temperature and blocked in 5% BSA. And the cells were incubated with an anti-STAT3 antibody, followed by incubation with FITC-conjugated anti-rabbit IgG (Santa Cruz) and PI for staining nuclei. Visualized on an IN Cell Analyzer 2000, image acquisition was configured to yield at least 1,000 cells per replicate well. Cytometric analysis performed with IN Cell Analyzer Workstation version 3.2. STAT3 nuclear entry was determined by measuring the nucleus/cytoplasm intensity ratio of green fluorescence with the Nuclear Translocation analysis module. Representatives of STAT3 nuclear translocation were shown as means ± SD.

### Statistical analysis

Statistical analysis was performed using a nonrepeated one-way analysis of variance followed by the Dunnett test for multiple comparisons. *p* values < 0.01 (two-tailed) were considered significant.

## Results

### Effects of stattic on everolimus-induced cell growth inhibition in various cell lines

**Figure 2:**
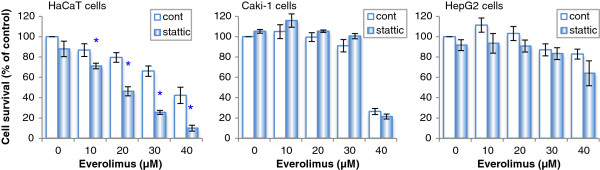
**Effects of a STAT3 inhibitor on the everolimus-induced cell growth inhibition in HaCaT, Caki-1, and HepG2 cells.** HaCaT, Caki-1, and HepG2 cells were incubated in medium containing everolimus at the indicated concentrations for 48 h after pretreatment with 10 μM stattic or DMSO (a solvent of stattic) for 20 min. Cell viability was determined by WST-8 colorimetric assay. *p < 0.01 Student’s t test compared with control (DMSO). There was no significant difference in cell toxicity in the DMSO, stattic, and 0 μM everolimus conditions for each cell line.

Figure 2 shows the everolimus-induced cell growth inhibition in HaCaT, Caki-1, and HepG2 cells in the absence or presence of the STAT3 inhibitor stattic. We found that the everolimus-induced cell growth inhibition in HaCaT cells was enhanced by pretreatment with stattic. In contrast, the everolimus-induced cell growth inhibition in Caki-1 and HepG2 cells was unaffected by stattic treatment. There was no significant difference on absorbance values with cell toxicity of control and stattic as not including everolimus in these cells.

### Effects of STAT3 inhibitors on apoptotic effects in HaCaT cells

**Figure 3:**
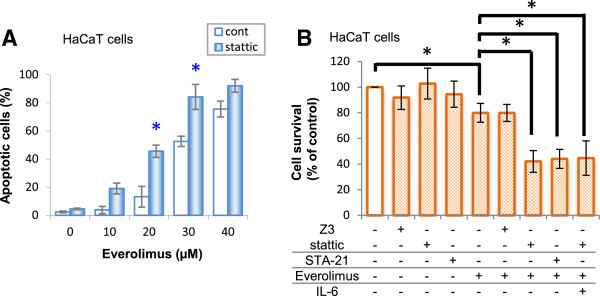
**Effects of various STAT3 pathway inhibitors on everolimus-mediated apoptotic effects and cell growth inhibition in HaCaT cells. (A)** HaCaT cells were incubated in medium containing everolimus at the indicated concentrations for 48 h after pretreatment with 10 μM stattic or DMSO for 20 min. Subsequently, apoptotic cells were detected using FITC-labeled Annexin V/PI staining on an IN Cell Analyzer 2000 for Imaging cytometric analysis. **(B)** Effects of JAK/STAT pathway inhibitors and IL-6 on the cell growth inhibition induced by everolimus. HaCaT cells were incubated in medium containing 30 μM everolimus for 48 h after pretreatment with 10 μM stattic for 20 min or coincubation with everolimus and 25 μM Z3 (a selective inhibitor of JAK2), 20 μM STA-21, 100 ng/mL IL-6, or DMSO (solvent of these inhibitors). Cell viability was determined by WST-8 colorimetric assay.

To confirm that the apoptotic effects of everolimus were enhanced by pretreatment with stattic, we performed an apoptosis assay (Figure 3A). Imaging cytometric analysis of apoptotic cells by Annexin V/PI staining showed that apoptosis in HaCaT cells was increased after everolimus treatment in a dose-dependent manner. Moreover, the percentage of apoptotic cells was enhanced by stattic pretreatment. These results indicate that stattic pretreatment enhances the apoptotic effects of everolimus in HaCaT cells.

### Effects of various JAK/STAT pathway inhibitors on everolimus-induced cell growth inhibition in HaCaT cells

In the presence of another STAT3 inhibitor (STA-21), the everolimus-induced cell growth inhibition observed in HaCaT cells was also enhanced, whereas a JAK2 inhibitor (Z3) did not affect the everolimus-induced cell growth inhibition (Figure 3). This synergistic cell growth inhibition effect was not due to coincubation with IL-6.

### Effects of everolimus and STAT3 inhibitors on signal transduction in HaCaT cells

**Figure 4:**
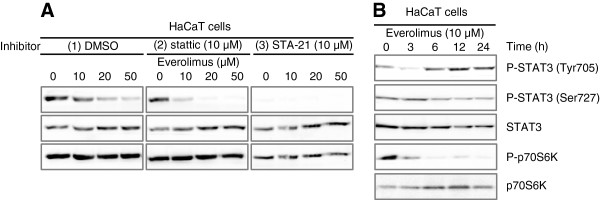
**Effects of various STAT3 inhibitors on everolimus-mediated signal transduction in HaCaT cells. (A)** Alteration in signal transduction of STAT3. HaCaT cells were incubated in medium containing everolimus at the indicated concentrations for 2 h (1): after pretreatment with 10 μM stattic for 20 min or (2): coincubation with everolimus and 10 μM STA-21 or (3) vehicle alone (DMSO). **(B)** Alteration in signal transduction of STAT3. HaCaT cells were incubated in medium containing 10 μM everolimus at the indicated time. Total cell lysates were separated by SDS-PAGE and electrotransferred to PVDF membranes. Various proteins and phosphorylation levels were evaluated by immunoblotting assay with specific antibodies.

Signal transduction in the presence of everolimus and pretreatment with stattic in HaCaT cells is shown in Figure 4. Phosphorylation of Tyr705 of STAT3 was decreased after treatment with everolimus for 2 h in a dose-dependent manner in HaCaT cells. In contrast, phosphorylation of Ser727 of STAT3 was unaffected by everolimus treatment in HaCaT cells in the absence of stattic; however, it increased slightly in the presence of stattic. Tyr705 phosphorylation was decreased by treatment with everolimus in the presence of pretreatment with stattic. Moreover, to clarify how STAT3 and mTOR regulate cell toxicity whether in a parallel manner or in a downstream regulation, we examined if STAT3 activity varies in a time-dependent manner with treatment of everolimus (Figure 4B). Phosphorylation of STAT3 was decreased in short-term but increased in long-term incubated with low-dose everolimus. Phosphorylation of p70 S6K which is direct downstream of mTORC1 showed inhibition in a time-dependent manner based on the mechanism of action of everolimus. This results show that STAT3 phosphorylation can be regulated indirectly by mTOR.

### Effects of everolimus on MAPKs activity in HaCaT cells and effects of MAPK inhibitors on everolimus-induced cell growth inhibition in HaCaT cells

**Figure 5:**
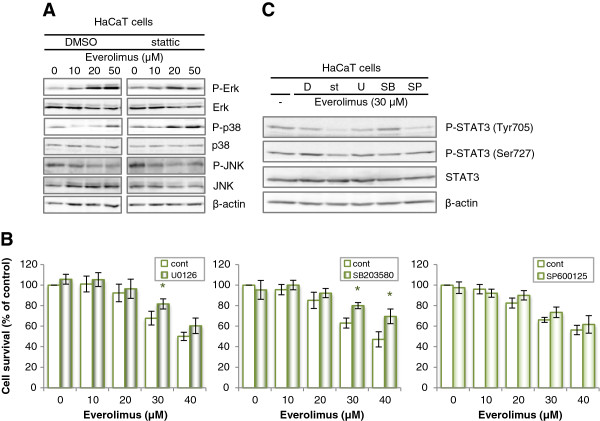
**Effects of everolimus on MAPKs activation in HaCaT and effects of MAPK inhibitors on everolimus-induced cell growth inhibition and signal transduction. (A)** Alterations in the signal transduction of MAPKs. HaCaT cells were incubated in medium containing everolimus at the indicated concentrations for 2 h after pretreatment with 10 μM stattic or DMSO. Total cell lysates were separated by SDS-PAGE and electrotransferred to PVDF membranes. Various proteins and phosphorylation levels were evaluated by immunoblotting assay with specific antibodies. **(B)** Effects of MAPK inhibitors on everolimus-induced cell growth inhibition. HaCaT cells were incubated with medium containing everolimus at the indicated concentrations for 48 h after pretreatment with U0126 (a MEK1/2 inhibitor, 10 μM) for 2 h, SB203580 (a p38 MAPK inhibitor, 10 μM) for 1 h, SP600125 (a JNK inhibitor, 20 μM) for 30 min, or DMSO (their solvent) for 2 h. Cell viability was determined by WST-8 colorimetric assay. *p < 0.01 Student’s t test compared with control (DMSO). Each bar represents the mean ± SD (n = 4). **(C)** Alterations in the signal transduction of STAT3 in the presence of MAPKs inhibitor. HaCaT cells were incubated in medium containing 30 μM everolimus for 2 h after pretreatment with 10 μM stattic for 20 min (st), 10 μM U0126 for 2 h (U), 10 μM SB203580 for 1 h (SB), 20 μM SP600125 for 30 min (SP) or DMSO (D). Total cell lysates were separated by SDS-PAGE and electrotransferred to PVDF membranes. Various proteins and phosphorylation levels were evaluated by immunoblotting assay with specific antibodies.

Previous studies demonstrated that the PI3K/Akt/mTOR and MAPK pathways represent a cross-linked signal network in various cell lines, and that STAT3 is an important downstream signaling factor of these pathways [25-27]. Therefore, we confirmed the differences in the phosphorylation of JNK, Erk1/2, and p38 MAPK after treatment with everolimus in HaCaT cells (Figure 5A). The phosphorylation of Erk1/2 and p38 MAPK was increased after treatment with everolimus in a dose-dependent manner in HaCaT cells. Moreover, the phosphorylation of p38 MAPK was particularly increased in the presence of pretreatment with stattic. Figure 5B shows the everolimus-induced cell growth inhibition in HaCaT cells in the absence or presence of a MEK1/2 inhibitor (U0126), a p38 MAPK inhibitor (SB203580) or a JNK inhibitor (SP600125). Treatment with the p38 MAPK inhibitor reduced the efficacy of cell growth inhibition by everolimus in HaCaT cells. A MEK1/2 inhibitor also affect the everolimus-induced cell growth inhibition in HaCaT cells, slightly. Moreover, we examined a possibility that MAPKs inhibitors rescue the inhibition of phosphorylation of STAT3 by everolimus (Figure 5C). In the pretreatment of SB203580, STAT3 Tyr705 phosphorylation was enhanced comparing from treatment of everolimus alone.

### Effects of STAT3 Y705F and STAT3C transfection on everolimus-induced cell growth inhibition in HaCaT cells

**Figure 6:**
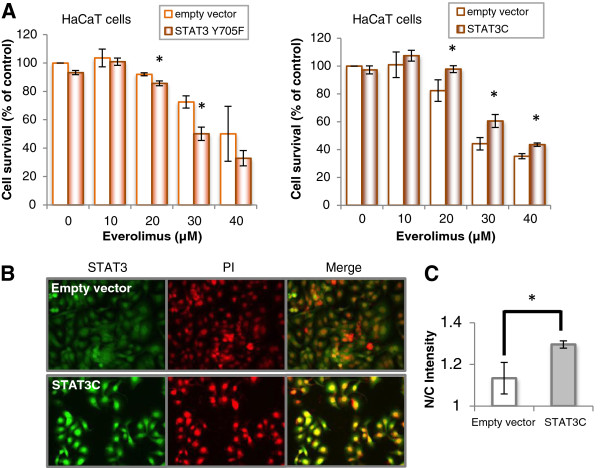
**Effects of dominant negative and constitutively active STAT3 on everolimus-induced cell growth inhibition in HaCaT cells. (A)** Effects of STAT3 Y705F and STAT3C transfection on everolimus-induced cell growth inhibition. HaCaT cells transiently transfected with STAT3 Y705F, STAT3C or each empty vector were incubated in medium containing everolimus at the indicated concentrations for 48 h after preincubation for 24 h. Cell viability was determined by WST-8 colorimetric assay. *p < 0.01 Student’s t test compared with control (DMSO). There was no significant difference in the cell toxicity between the empty vector and STAT3C transfection. **(B)** Immunostaining images. HaCaT cells transiently transfected with STAT3C or empty vector were fixed and incubated with an anti-Stat3 antibody, followed by incubation with FITC-conjugated anti-rabbit IgG (green) and visualization on an IN Cell Analyzer 2000. **(C)** STAT3 nuclear entry was determined by measuring the nucleus/cytoplasm intensity ratio of green fluorescence (n = 3). *p < 0.05 Student’s *t* test compared with control.

STAT3C is a constitutively active STAT3 that dimerizes constantly by substituting cysteine residues for specific amino acids within the C-terminal loop of the STAT3 molecule [23], which resulted in the assembly of STAT3 in the nucleus of transfected cells (Figure 6B and C). Transfection of cells with STAT3 Y705F had a tendency to enhance the cellular toxicity of everolimus compared with transfection with an empty vector, but STAT3C had a tendency to relieve, as shown in Figure 6A.

## Discussion

A recent study reported that common cutaneous dermatological side effects develop after treatment with EGF receptor (EGFR) inhibitors (e.g., cetuximab, panitumumab, and erlotinib), mTOR inhibitors (e.g., everolimus and temsirolimus), and multikinase inhibitors (e.g., sorafenib and sunitinib) [1-5,7-9,28-30]. These drugs exert a beneficial effect by inhibiting a close line of signal transduction; therefore, we thought that the key factor involved in the dermatological events observed may be a downstream factor converging from PI3K and MAPK pathways. STAT3 is activated by stimulation from PI3K, MAPK, and JAK2 pathways; thus, we hypothesized that STAT3 is a candidate factor for regulating dermatological events induced by molecular target drugs.

Cell growth inhibition by everolimus in HaCaT cells was enhanced by pretreatment with STAT3 inhibitors (stattic and STA-21), but not by pretreatment with a JAK2 inhibitor (Figures 2 and 3B). We interpreted this phenomenon in the following manner: the everolimus-induced cell growth inhibition involved in STAT3 in keratinocytes, depends on signaling from growth factors, i.e., PI3/Akt or MAPK pathways, and not on the IL-6/JAK2 pathway. Everolimus and STAT3 inhibitors inhibited cell growth synergistically and increased the number of apoptotic cells (Figure 3A), but there was a little difference between the survival data and the apoptosis data. A cause of this difference considered that treatment time between cell survival analysis and apoptosis analysis was differed. In the cell survival analysis, each cell was treated with everolimus for 48 h, but in the apoptosis analysis, HaCaT cells were incubated with everolimus for 24 h, because it was necessary that cell spacing be got at the point of measurement to evaluate apoptosis marker appropriately in imaging cytometric analysis. Incubating for 48 h in control cells could not get adequate cell spacing. Moreover, STAT3 activation is suggested to differ between human immortalized keratinocyte HaCaT cells and normal human keratinocytes [31]. We confirmed that everolimus-induced cell growth inhibition was enhanced by STAT3 inhibition in normal human epidermal keratinocyte NHEK cells (data not shown). Because similar results were obtained in our study using NHEK cells, we suggest that the same phenomenon may occur in normal keratinocyte cells characterized of having less STAT3 activity. In addition, our study showed that cell survival differed in each cell type in the presence of STAT3 inhibitors. This suggests that stattic behaved similarly in each cell line, but may differ greatly depending on cell types that contributing rate of STAT3 in the cell survival.

Another recent study reported that cooperation of the two phosphorylated residues is necessary for the full activation of STAT3 [31-34]. In our study, Tyr705 phosphorylation was decreased by treatment with everolimus in a dose dependent manner in short-term treatment, however in long-term for 12–24 h, Tyr705 phosphorylation increase by treatment with low-concentration everolimus in HaCaT cells. Ser727 phosphorylation was not decreased, rather, it was slightly increased in short-term treatment, but in long-term for 12–24 h, Ser727 phosphorylation decrease by treatment with low-concentration everolimus (Figure 4). Stattic inhibits Tyr705 phosphorylation and the dimerization of STAT3 molecules, and Ser727 phosphorylation should not be affected by stattic [16]. This results show that Tyr705 phosphorylation can be regulated indirectly by mTOR. It is known that a mTOR inhibitor cause compensatory activation of MAPKs signal [35,36]. And, It is also known that MAPKs regulate STAT3 activity, therefore, we considered that the inhibition of phosphorylation of STAT3 by everolimus mediate MAPKs pathway.

It is well known that the STAT3 Ser727 residue is phosphorylated mainly by Erk1/2, p38 MAPK, JNK and mTOR [37-40]. Our results showed that everolimus activated Erk and p38 MAPK and phosphorylated STAT3 at Ser727, which SB203580 inhibited phosphorylation of STAT3 at Ser727 (Figures 4 and 5). A negative effect of Ser727 phosphorylation on Tyr705 phosphorylation in STAT3 has also been suggested [41]. These results support those of previous reports showing that activated Erk and p38 may synergistically regulate STAT3 activity in a negative manner. In addition, although JNK did not affect everolimus-mediated cell growth inhibition, the p38 MAPK inhibitor depressed everolimus-induced cell growth inhibition in HaCaT cells (Figure 5). The phosphorylation of p38 MAPK was increased by exposure to everolimus, and inhibition of phosphorylation of STAT3 Tyr705 by everolimus rescued by pretreatment of SB203580. mTOR inhibition by everolimus results in inhibition of de novo protein synthesis, and results in p38 MAPK activation due to sense cellular stress, moreover they may result in STAT3 inhibition [35]. We considered that p38 MAPK may be largely involved in the everolimus-induced inhibition of STAT3 activity in keratinocytes. So, Erk phosphorylation was also activated by everolimus and U0126 depressed everolimus-induced cell growth inhibition slightly in HaCaT cells. It is well known that Erk regulate STAT3 activity negatively [38]. Erk activity may partially contribute to everolimus-induced cell growth inhibition in keratinocyte. p38 MAPK pathways are known as stress response signals and interact with the PI3K/Akt/mTOR pathway [36]. Recently, it was reported that keratinocyte apoptosis induced by gefitinib, which is a selective EGFR tyrosine kinase inhibitor, is mediated by the JNK activation pathway [42]. This study did not reproduce the results of that report; therefore, the mechanisms underlying everolimus-induced keratinocyte apoptosis may differ from those underlying gefitinib-induced apoptosis. Alternatively, they may be one of the gefitinib-induced mechanisms because the gefitinib target signal lies upstream from the target of everolimus.

In addition, because STAT3 Y705F enhanced cell toxicity in HaCaT cells and STAT3C relived, the survival of this type of keratinocytes may depend largely on STAT3 (Figure 6). For comparison, we considered that an active form of STAT3 subtly rescued everolimus-induced toxicity because cell temporary transfection efficiency of pcDNA3 STAT3C with lipofection method in HaCaT cells was not higher as a result of confirming STAT3 expressions with western blotting assay. To corroborate this effects of rescue by STAT3C, it’s necessary in the future to conduct an experiments with HaCaT cells stably expressed STAT3C.

Previous reports have suggested that STAT3 inhibition in cutaneous squamous cell carcinoma induces senescence and not apoptosis [43]. Though apoptosis suppressing genes (e.g., bcl-2) and senescence factors (e.g., AP-1) were not evaluated in our study, both apoptotic and senescent effects may have affected the cell growth inhibition induced by everolimus and the STAT3 inhibitor. In addition, the apoptotic effects observed in our study may have been enhanced by interaction with the effects of mTOR and STAT3 inhibition.

Everolimus is distributed by P-glycoproteins and metabolized by CYP3A4 [44,45]. Although the pharmacokinetic profiles of stattic have not been clarified, there is no denying that the interactions between everolimus and stattic are due to pharmacokinetic actions. We have previously demonstrated that calcium antagonists and α-adrenoceptor antagonists enhanced cellular sensitivity to SN-38, an active metabolite of irinotecan, by increasing the concentration of SN-38 in cells [21,22]. It is difficult to assume that a similar phenomenon caused the effects observed in this study; however, the involvement of STAT3 may be the greater part of this interaction because a similar phenomenon was caused by STA-21, which has a chemical structure that is different from that of stattic, and STAT3C transfection moderated everolimus-induced cell growth inhibition.

In clinical practice, it is known that the efficacy of molecular target drugs is correlated with their toxicity. It has been reported that inhibition of STAT3 by sunitinib contributes to the induction of apoptosis in renal cell carcinoma [46]. Moreover, STAT3 is known to have functional single nucleotide polymorphisms (SNPs). These SNPs have been reported to be predictive tools for the efficacy of IFN treatment against metastatic renal cell carcinoma [47]. Based on these reports and the present study, we hypothesized that STAT3 would be a critical factor for the treatment of renal cell carcinoma and toxicity to skin tissue, and that responsibility of STAT3 depend on functional SNPs. However, it remains unclear that the everolimus-induced cell growth inhibition in Caki-1 and HepG2 cells was unaffected by stattic treatment. SNPs genotyping analysis of STAT3 in various cells is required to address these issues in the future. In addition, through our research, patients carrying a high risk of dermatological toxicity by molecular target drugs could be identified by testing for STAT3 polymorphisms. And, ultraviolet (UV) irradiation increases the potential of dermatological side effects induced by molecular target drugs in clinical reports [48]. STAT3 represents a critical regulator of keratinocytes in response to UVB irradiation [49]. After UVB irradiation, STAT3 is rapidly downregulated in keratinocytes, which leads to decreased cell cycle progression and increased sensitivity to UVB-induced apoptosis. It has also been reported that UV specifically decreases the DNA binding activity of STAT3 [50]. Furthermore, UV triggers the activation of members of the MAPK family, including Erk1/2, JNK, and p38 MAPK [50]. UV irradiation can enhance MAPK activity and lead to a greater phosphorylation of STAT3 at Ser727 in the presence of everolimus [26,51]. These results suggest that the dermatological side effects induced by molecular target drugs can be increased potentially by UV irradiation, with repression of STAT3 activity mediating greater phosphorylation of Ser727. However, additional studies are necessary to clarify this potency.

## Conclusions

In conclusion, STAT3 activation may be a key factor in everolimus-induced keratinocyte cytotoxicity. Moreover, p38 MAPK and Erk mediated between mTOR signaling and STAT3 signaling may also play an important role of everolimus-induced dermatological side effects. Skin reactions caused by everolimus or other molecular target drugs may cause significant physical discomfort, thus decreasing the quality of life of patients or leading to the discontinuation of drug therapy. Therefore, a mechanism-based approach, and not just clinical experience-based treatment strategies, to assess dermatological toxicity should be proposed to overcome this uncomfortable reaction. We advocate that cutaneous localized treatment aimed at the maintenance of the homeostasis of STAT3 activity may be an effective strategy.

## Competing interests

The authors declare that they have no competing interests.

## Authors’ contributions

KY carried out the molecular genetic studies and drafted the manuscript. AU and AM performed the statistical analysis. KY, TH, MK, HM and TB participated in its design and coordination. TB, CN, MH helped to draft the manuscript. All authors read and approved the final manuscript.
